# Lipopolysaccharide Renders Transgenic Mice Expressing Human Serum Amyloid P Component Sensitive to Shiga Toxin 2

**DOI:** 10.1371/journal.pone.0021457

**Published:** 2011-06-24

**Authors:** Thomas P. Griener, Jonathan G. Strecker, Romney M. Humphries, George L. Mulvey, Carmen Fuentealba, Robert E. W. Hancock, Glen D. Armstrong

**Affiliations:** 1 Department of Microbiology, Immunology and Infectious Diseases, Faculty of Medicine, University of Calgary, Calgary, Alberta, Canada; 2 Faculty of Veterinary Medicine, University of Calgary, Calgary, Alberta, Canada; 3 Department of Microbiology and Immunology, University of British Columbia, Vancouver, British Columbia, Canada; 4 Department of Pathology and Laboratory Medicine, University of California Los Angeles, Los Angeles, California, United States of America; Institut Pasteur, France

## Abstract

Transgenic C57BL/6 mice expressing human serum amyloid P component (HuSAP) are resistant to Shiga toxin 2 (Stx2) at dosages that are lethal in HuSAP-negative wild-type mice. However, it is well established that Stx2 initiates extra-intestinal complications such as the haemolytic-uremic syndrome despite the presence of HuSAP in human sera. We now demonstrate that co-administering purified *Escherichia coli* O55 lipopolysaccharide (LPS), at a dosage of 300 ng/g body weight, to HuSAP-transgenic mice increases their susceptibility to the lethal effects of Stx2. The enhanced susceptibility to Stx2 correlated with an increased expression of genes encoding the pro-inflammatory cytokine TNFα and chemokines of the CXC and CC families in the kidneys of LPS-treated mice, 48 hours after the Stx2/LPS challenge. Co-administering the glucocorticoid dexamethasone, but not the LPS neutralizing cationic peptide LL-37, protected LPS-sensitized HuSAP-transgenic mice from lethal doses of Stx2. Dexamethasone protection was specifically associated with decreased expression of the same inflammatory mediators (CXC and CC-type chemokines and TNFα) linked to enhanced susceptibility caused by LPS. The studies reveal further details about the complex cascade of host-related events that are initiated by Stx2 as well as establish a new animal model system in which to investigate strategies for diminishing serious Stx2-mediated complications in humans infected with enterohemorrhagic *E. coli* strains.

## Introduction

Haemolytic-uremic syndrome (HUS) is characterized by a triad of clinical signs including thrombocytopenia, microangiopathic haemolytic anaemia, and acute renal failure. Typically, HUS occurs in young children as a consequence of an infection by enterohemorrhagic *Escherichia coli* (EHEC) strains, including, but not limited to, O157:H7 serotype strains. HUS is generally thought to be initiated when the Shiga toxins, which are expressed by EHEC, cause injury to endothelial cells lining the glomerular capillaries. Although Shiga toxin 2 (Stx2) is more commonly associated with HUS than Stx1 [1], [2], immunoglobulin-depleted human serum possesses an innate ability to neutralize the cytotoxic activity of Stx2 [3]. The factor responsible for this neutralizing activity was identified by Kimura *et al.* as human serum amyloid P component (HuSAP) [4].

HuSAP is a member of the pentraxin protein family and acts as a soluble pattern recognition molecule which interacts with altered self-antigens and conserved microbial components subsequently targeting these for catabolic disposal *via* the reticuloendothelial system [5], [6]. We previously demonstrated that HuSAP also inhibits Stx2 from binding its cellular receptor, the glycolipid globotriaosylceramide (Gb_3_) [7]. This activity is apparently unique to HuSAP, since serum from a variety of other species, including mice, fails to neutralize Stx2 [4]. We further demonstrated that exogenous or endogenous HuSAP protects mice challenged with purified Stx2 [8]. In contrast, we also showed that the initial toxin-mediated renal damage in humans can occur despite the presence of normal concentrations of HuSAP in their sera.

Although injecting purified Stx2 containing little LPS into mice results in acute renal failure, one of the three clinical signs of HUS, the renal pathology in mice is distinct from that observed in human cases of HUS. However, injecting a low dose of *E. coli* O55 LPS, along with a single lethal dose of Stx2, was reported to reproduce many of the human signs of HUS in mice including neutrophilia, increased serum creatinine and blood urea nitrogen (BUN) and most notably, glomerular fibrin deposition and endothelial damage [9]. Interestingly, Gb_3_ is not expressed in the murine glomerulus and Stx2 specifically damages the murine collecting duct epithelia in this model [10]. The reason for the glomerular damage observed in LPS-treated Stx2-challenged mice is therefore unclear; however similar glomerular damage has also been observed when mice are injected with multiple low doses of LPS-free Stx2, indicating that LPS is not required to elicit such damage [11]. Regardless, many of the immunological changes observed in EHEC-infected HUS patients were also observed in mice receiving both LPS and purified Stx2.

In subjects with HUS, there is abundant evidence of an acute inflammatory response, suggesting that at least low-grade endotoxemia may occur following EHEC infection, despite the characteristic absence of fever [12]. HUS patients often exhibit a systemic cytokine response with increased serum concentrations of pro-inflammatory cytokines such as interleukin (IL)-8, IL-1β, IL-6 and, in some cases, tissue necrosis factor α (TNFα), as well as a decline in anti-inflammatory cytokines [13], [14], [15], [16], [17], [18]. The presence of these cytokines has also been detected in urine of some HUS patients, suggesting that a local inflammatory response in the kidneys may occur as well [17], [18], [19]. These findings are consistent with the observation of neutrophil activation and accumulation in the glomeruli of children with HUS, and an associated elevation in CXC chemokines such as growth-related oncogene (CXCL1/GRO) and granulocyte colony stimulating factor (G-CSF) [16], [20], [21]. Increased serum concentrations of monocyte chemotactic protein 1 (CCL2/MCP-1), macrophage inflammatory protein 1 (CCL4/MIP-1) and renal macrophage infiltration have also been reported in humans [19], [20]. These chemokines appear to play an important role in pathology since the level to which they are increased positively correlates with the severity of the renal damage.

For the most part, the changes in cytokine and chemokine concentrations in humans are reproduced in mice when LPS and Stx2 are co-administered. The circulating concentrations of IL-6 and TNF-α increase dramatically immediately following injection of Stx2 and LPS, as does the production of chemokines CCL2, CCL3 (MIP-1), and CXCL1 in the murine kidney [9], [22], [23], [24]. Coupled with increases in vascular cell adhesion molecule 1 (VCAM-1) and intercellular adhesion molecule 1 (ICAM-1), these changes appear to facilitate the glomerular infiltration of neutrophils and macrophages and contribute to the observed renal damage [9], [22], [24]. Furthermore, Stx2 and LPS treatment activates coagulation/fibrinogenesis pathways, as indicated by increased plasma thrombin-antithrombin III (TAT) complex, fibrinogen and plasminogen activator inhibitor-1(PAI-1) levels and decreased platelet counts [9], [23], all of which have also been detected in human HUS patients [25], [26], [27]. The observation that co-administering LPS and Stx2 shifted the spectrum of pathological changes in mice to one more closely resembling that observed in HUS victims prompted us to investigate whether LPS would also alter the susceptibility of HuSAP transgenic mice to Stx2 and the results of these investigations are reported herein.

## Methods

### LPS-sensitized mouse Shigatoxemia model

All animal work was conducted according to the guidelines of the Canadian Council on Animal Care with the approval of the University of Calgary Health Sciences Animal Care Committee (Protocol #M07054). The transgenic mice used for these experiments were C57BL/6-Tg(APSC)1Imeg mice, which exhibit liver-specific expression of HuSAP at a stable circulating serum concentration of 30–40 µg/mL, similar to the concentration in human serum. These mice were first created by Zhao *et al.* and were bred in-house [28]. Stx2 was affinity purified using Synsorb-P^K^ and residual LPS was removed using Endotrap blue LPS affinity columns as described previously [29]. The concentration of LPS in the purified Stx2 preparations was determined to be less than 0.001 endotoxin units per µg protein as determined by the colorimetric Limulus amebocyte lysate assay. Control wild-type (WT) and transgenic HuSAP+ C57BL/6 mice (male and female, age 8–10 weeks) were IP injected with 225 pg/g body weight (BW) Stx2 alone, 300 ng/g BW of *E. coli* O55 LPS (Sigma-Aldrich) alone, or both according to the protocol described by Keepers *et al.* [9].

Five µL of blood was drawn from the tail vein prior to and 10 hours after the injections and diluted 1∶8 in crystal violet diluent (0.1 mg/mL crystal violet, 1% (v/v) acetic acid) to lyse red blood cells and stain remaining leukocytes. White blood cells were enumerated using a haemocytometer. Blood smears were also prepared from these samples. These were methanol-fixed and then Wright-Giemsa stained for differentiation of individual cell types (HARLECO Hematology Stain, EMD Chemicals, Gibbstown, New Jersey). Mice were euthanized by CO_2_ asphyxiation at the onset of clear signs (lethargy) of Shigatoxemia and their kidneys were removed and fixed overnight in 10% buffered (pH 7.2–7.4) formalin prior to paraffin embedding. Four micron sections were stained with hematoxylin and eosin (H&E), as well as periodic acid-Schiff (PAS), and phosphotungstic acid-hematoxylin (PTAH). Animals that had not succumbed to the effects of Stx2 by 120 hours were euthanized and their kidneys were removed for light microscopy examination. Microscopic examination was performed on sagittal sections of the kidney that included the papilla, cortex and medulla. Histopathological changes were described according to distribution, severity and morphological features and were scored on a scale of 0–4 (0 = no observable abnormalities, 1 = minimal or less than 10% of tissue affected; 2 = Mild or 11 to 25% of tissue affected; 3 = Moderate or 26 to 50% of tissue affected; 4 = Severe or more than 50% of tissue affected). The pathologist (CF) was unaware of the treatment groups.

In a separate experiment, HuSAP-negative (WT) C57BL/6 mice (Charles River Laboratories International Inc., Wilmington, Massachusetts) were IP injected with 6.3, 20, 63 or 200 pg/g BW of Stx2 alone or Stx2 admixed with a constant 300 ng/g BW of LPS.

### Stx2 Vero cytotoxicity neutralization assay

Stx2 cytotoxicity in the presence and absence of HuSAP and LPS was determined using the Vero cytotoxicity assay. HuSAP (supplied by Dr. Mark Pepys, University College London, United Kingdom) was incubated in 100 µL minimal essential medium (MEM) at 20 µg/mL (or 3-fold serial dilutions beginning from 20 µg/mL) for 30 minutes at 37°C+5% CO_2_ either alone or admixed with 3-fold serial dilutions of *E. coli* O55 LPS (starting from 20 µg/mL). Stx2 (20 ng/mL) in 100 µL MEM+10% fetal bovine serum (FBS) was then added and the mixture was incubated for 1 h at 37°C+5% CO_2_, before the entire volume (200 µL) was transferred to confluent Vero cell monolayers in 96 well tissue culture plates. The Vero cells were incubated for 2 hours and the media containing toxin was then removed. After gently washing the monolayers with MEM two times, 200 µL of MEM+10% FBS was added and the cells were incubated at 37°C+5% CO_2_ for 72 hours. Surviving cells were fixed with 10% methanol and Giemsa-stained and absorbance was measured to assess Vero cell survival. To test the ability of LPS to inhibit HuSAP-mediated neutralization of Stx2 in the presence of other blood components such as LPS-binding protein, serial dilutions of serum from a healthy human volunteer were incubated with Stx2 (20 ng/mL) for 1 h at 37°C+5% CO_2_ prior to adding to Vero cell monolayers. Serum was incubated with or without LPS (100 µg/mL) for 30 min prior to incubating with Stx2.

### HuSAP-Stx2 ELISA

Stx2 binding to HuSAP in the presence and absence of LPS was determined using a solid-phase ELISA-type binding assay. Microtitre plates (Corning Incorporated, Lowell, Massachusetts) were coated with purified Stx2 holotoxin at a concentration of 2 µg/mL in 10 mM Tris and 0.14 M NaCl, pH 8.0 buffer (TN buffer) or TN buffer containing 2 mM CaCl_2_ (TC buffer) and then thoroughly washed five times with TN or TC buffer before blocking with 3% (w/v) bovine serum albumin (BSA – Thermo Scientific, Waltham, Massachusetts) in TN or TC buffer. A constant concentration of 20 µg/mL of HuSAP alone, or admixed with 100 µg/mL LPS, in TN or TC buffer supplemented with 4% (w/v) BSA (prevents non-specific aggregation of HuSAP, M. B. Pepys, personal communication) was added to the 96 well microtiter plates which were then incubated for an additional 1 h. The microtiter plates were again thoroughly washed (five times with TN or TC buffer +4% BSA). Bound HuSAP was detected using a 2000 fold dilution of polyclonal rabbit anti-HuSAP antibody (Dako Canada Inc., Mississauga, Ontario) followed by a 2000 fold dilution of monoclonal goat anti-rabbit IgG antibody conjugated to horseradish peroxidase (Sigma-Aldrich), both in TN or TC buffer. Peroxidase was detected using the colourimetric 2,2′-azino-bis(3-ethylbenzthiazoline-6-sulphonic acid) reagent (ABTS, Sigma-Aldrich) in 50 mM sodium citrate buffer (pH 4.2). Absorbance at 410 nm was recorded using a SpectraMAX340 plate reader (Molecular Devices, Sunnyvale, California) after 20 min development.

### Inflammatory response to Stx2 and LPS

Mice (WT and HuSAP+) were IP injected with Stx2 (225 pg/g BW) and/or LPS (300 ng/g BW). After 2 or 48 h, the mice were euthanized by CO_2_ asphyxiation and blood was collected by cardiac exsanguination into heparin-containing tubes. The kidneys were removed and 30 mg portions were stored at −20°C in RNA*later*® solution (Applied Biosystems, Austin, Texas). RNA was purified from stored tissue using the RNeasy Mini Kit using the protocol recommended by the manufacturer (Qiagen Inc. Valencia, California). Tissue was homogenized in lysis buffer using a Polytron 1300D homogenizer and tissue debris was subsequently removed by centrifugation. RNA was eluted from the column in RNAse-free water and, to ensure accuracy in the detection of potentially low copy mRNA transcripts, genomic DNA was removed with the DNA-*free™* kit (DNAse I digestion) according to the manufacturer's instructions (Ambion, Austin, TX, USA). Purified RNA was used to generate cDNA using the First Strand Synthesis kit (Invitrogen). Briefly, cDNA was generated from 1 µg of RNA by reverse transcription using oligo(dT)_20_ primers and SuperScript™ II Reverse Transcriptase (RT). The remaining RNA was degraded using RNAse H.

Expression of pro-inflammatory cytokine, chemoattractant and coagulation factor genes was assessed by quantitative real-time PCR (qPCR) of various targets which were selected for analysis based on the results of previous studies [9], [22], [23], [24] where Stx2 and LPS were co-administered to WT mice, or based on their reported role in the development of HUS in EHEC-infected human subjects. The primers used for amplification and references for each selected gene are summarized in Table S1. Primers were first tested using traditional PCR and gel electrophoresis to ensure each primer set yielded a single product and gradient PCR was performed as required to identify suitable annealing temperatures. qPCR analysis was performed using an iQ5™ thermal cycler (Bio-Rad, Hercules, CA, USA) in 12.5 µL reactions using Maxima™ SYBR Green qPCR mix (Fermentas, Burlington, ON, Canada). Expression was normalized to the reference genes α-actin (*Actb*) and glyceraldehyde-3-phosphate dehydrogenase (*Gapdh*) by modifying the denominator of the Pfaffl equation which takes into account the relative efficiency of each primer pair [30]. PCR efficiency was calculated by a limiting dilution experiment, in which five 10-fold serial dilutions were examined (Table S1). Melting curves were performed on all qPCR reactions to ensure specific products were generated and all samples with incorrect or multiple peaks were not analyzed further. Each mouse cDNA sample was analyzed for each gene in triplicate to account for template loading and amplification inconsistencies. The quantification cycles in triplicate were averaged for each mouse and compared to the no treatment group to determine a relative fold change in gene expression. The relative fold increase in gene expression was then averaged within each group with standard deviation indicating the variation between the three mice.

Serum IL-6 concentrations in Stx2-challenged LPS-treated mice were determined using the OptEIA capture ELISA (BD Biosciences) method. 96 well microtitre plates were coated overnight with a 250-fold dilution of anti-mouse IL-6 capture antibodies in PBS. The microtiter plates were washed three times with PBST and then blocked for two hours with PBS+10% FBS. The microtiter plates were again washed three times with PBST. Two-fold serial dilutions of mouse serum samples, as well as recombinant mouse IL-6 standards, were prepared in PBS+10% FBS and added to the microtiter plates. After two hours, the plates were thoroughly washed seven times. A 250-fold dilution (in PBS+10% FBS) of biotinylated anti-mouse IL-6 detecting antibodies was added and the microtiter plates were incubated for 1 h. The plates were again washed five times and a 250-fold dilution (in PBS+10% FBS) of avidin-HRP detecting reagent was added. The microtiter plates were incubated for 1 h, washed five times with PBST, and bound HRP was detected with ABTS reagent as previously described. Except for the anti-IL-6 coating step, which was performed at 4°C, all incubations were performed at 37°C. The resulting absorbance measurements were used to calculate the concentration of each cytokine in the initial serum samples by comparing to the standard curve generated using known concentrations of recombinant IL-6.

### Suppression of immune response and protection from Stx2/LPS challenge

Two compounds were tested for their ability to prevent the predominantly-LPS mediated inflammatory response in HuSAP+ mice and restore their resistance to Stx2. LL-37, a human cathelicidin-derived antimicrobial peptide, binds LPS with high affinity and prevents it from binding LPS binding protein and thereby activating immune cells [31]. In addition to this direct anti-endotoxin effect, LL-37 is able to modulate Toll-like receptor activation and pro-inflammatory gene transcription. Dexamethasone (DEX – Sigma-Aldrich Canada) is an anti-inflammatory glucocorticoid and has been shown previously to suppress the immune response to LPS [32], [33] and to provide some protection against Stx2 challenge [34]. For LL-37, HuSAP+ mice were IP injected with 225 pg/g BW Stx2 and 300 ng/g BW LPS either alone or admixed with 0.5 or 5.0 µg/g BW LL-37. For DEX, HuSAP+ or WT mice were subcutaneously injected with 2.5 µg/g BW DEX or control saline 30 minutes prior to the IP injection of Stx2 and LPS, according to a previously published protocol for LPS neutralization [32]. Mice were weighed and monitored for signs of Shigatoxemia over a period of 150 h. In a separate experiment, HuSAP+ mice were euthanized by CO_2_ asphyxiation and exsanguinated by cardiac puncture at 2 and 48 h for assessment of gene expression and serum cytokine concentrations as described in the previous section.

## Results

### LPS sensitizes HuSAP+ mice to Stx2

**Figure 1:**
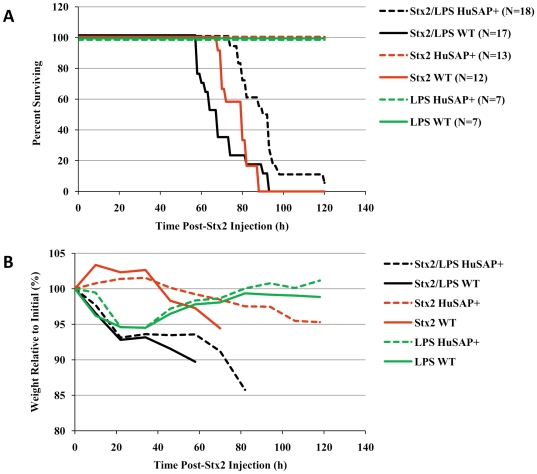
Survival and weight loss of HuSAP+ and WT mice following Stx2 and LPS challenge. HuSAP+ (*dashed lines*) mice and their WT littermates (*solid lines*) received one of three treatments. Mice were IP injected with 225 pg/g BW Stx2 in combination with 300 ng/g BW *E. coli* O55:H5 lipopolysaccharide *(black lines)*, 225 pg/g Stx2 alone *(red lines)*, or 300 ng/g LPS alone *(green lines)*. The number of animals in each group is listed beside each label. Animals were monitored every 2–4 hours, a representative group was weighed daily (**B**). Mice were euthanized by CO_2_ asphyxia if clear signs of lethargy were observed (**A**). The differences in survival between HuSAP+ that received Stx2 and those that received Stx2 and LPS was statistically significant (Log Rank test p<0.001). The difference in survival between HuSAP+ and WT mice were statistically significant (Log Rank test p<0.05) only in the absence of LPS.

**Figure 2:**
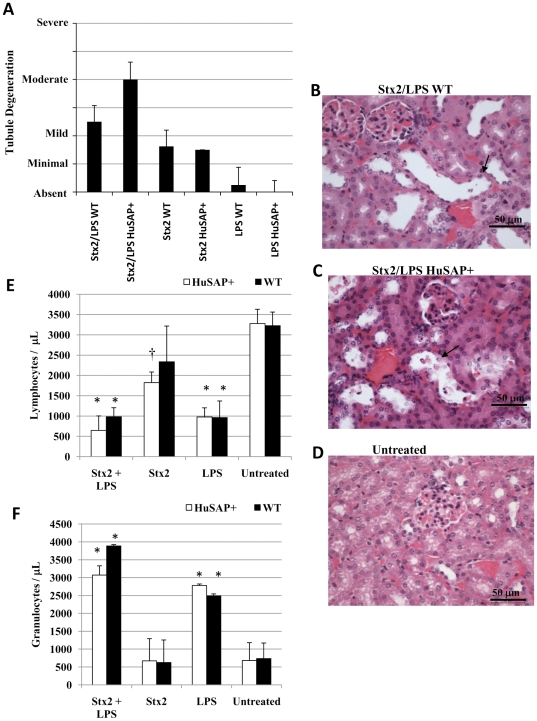
Morphologic changes following Stx2 and LPS challenge in HuSAP+ and WT mice. Kidneys were dissected from representative mice at the time of euthanasia as a result of Shigatoxemia or at 120 hours and were fixed in formalin before paraffin embedding and H&E staining. Consistent morphologic changes were restricted to the tubules in all groups and the severity of the tubular degeneration was scored and the results within each group averaged (**A**). In mice treated with Stx2 and LPS, the predominant changes in morphology were moderate tubular degeneration and dilatation (*solid black arrows*) with clear evidence of individual cell necrosis. The images presented are 40× magnification and are representative of the changes observed in all WT (**B**) and HuSAP+ (**C**) mice that were injected with Stx2 and LPS. A renal section from an untreated mouse is presented for comparison (**D**). The circulating lymphocyte (**E**) and neutrophil (**F**) numbers of toxin-challenged mice were determined 10 hours after toxin injection. Each bar represents the average of four mice in each group and the error bars represent the standard deviation. Asterisks (*) and crosses (†) indicate a significant difference (Student's *t*-test p<0.005 and p<0.05 respectively) relative to untreated mice.

As shown in Figure 1A, and consistent with our previous observations [8], HuSAP+ C57BL/6 transgenic mice are completely resistant to 225 pg/g BW purified Stx2, in contrast to WT mice which were completely sensitive (compare red dashed with solid red lines, Figure 1A, Log Rank/Mantel-Cox test p<0.001). However, injecting LPS in addition to Stx2 reversed the protective effect of HuSAP in these mice (black dashed line, Figure 1A). Like WT mice, HuSAP+ mice lost 5–10% of their initial body weight immediately (within 24 hours) following Stx2/LPS injection and all eventually succumbed to the combined challenge (Figure 1A, 1B). Importantly, none of the mice injected with LPS alone succumbed and minimal renal histological changes were detected in these animals (Figures 1 and 2).

HuSAP may still offer some protection since the average survival time (88.9+/−10.9 h) of the HuSAP+ mice challenged with Stx2 plus LPS was significantly (average time of death - Student's *t*-test p<0.001, Log Rank test p<0.001) longer relative to WT mice challenged with Stx2 plus LPS (70.1+/−12.4 h). However, the histological alterations observed in the kidneys of the mice at the time of euthanasia were generally more severe in HuSAP+ mice which received Stx2/LPS relative to WT mice which received the same treatment (Figure 2A). Consistent with the report by Keepers *et al.* [9], mice that received Stx2 plus LPS also exhibited greater signs, relative to mice receiving Stx2 alone, of neutrophilia and lymphocytopenia, two clinical signs characteristic of HUS, (Figure 2E and 2F) ten hours after toxin injection. These changes appeared to be mediated predominantly by LPS, since they occurred to a similar degree when LPS was injected alone. None of these symptoms were prevented or lessened by HuSAP. Therefore, despite its reported interaction with LPS [35], we found no evidence that HuSAP antagonizes LPS action in HuSAP+ C57BL/6 mice.

The histological appearance of renal tissue obtained from representative mice at the time of euthanasia, or from the survivors at 120 h, was generally consistent with the survival and weight loss observations (Figure 2A–D). Lesions were more severe in mice which received both Stx2 and LPS relative to those that received Stx2 alone. Histological examination revealed no obvious differences between the kidneys of mice treated with LPS alone relative to those of the untreated control mice. In contrast to the conclusions presented in a previous report that injecting Stx2 and LPS caused renal histopathology similar to that observed in human HUS patients [9], thrombosis was not observed in our Stx2/LPS-treated mice as revealed by both routine H&E and PTAH staining. The main histologic changes were restricted to the tubules and consisted of minimal to moderate tubular dilatation and degeneration with occasional individual necrotic epithelial cells and presence of necrotic debris within the lumen of dilated tubules. The results of this analysis are summarized in Figure 2A and representative images of WT and HuSAP+ mice that received Stx2 and LPS are presented in Figure 2B/C. The location of this damage is consistent with the cortical tubular epithelial cells identified by Psotka *et al.* as the site of Gb_3_ expression and Stx2-induced apoptosis [10]. Therefore, although LPS renders HuSAP+ mice sensitive to Stx2 challenge, these mice do not reproduce the histopathological alterations associated with HUS in humans.

### Effect of LPS on HuSAP-Stx2 Binding

**Figure 3:**
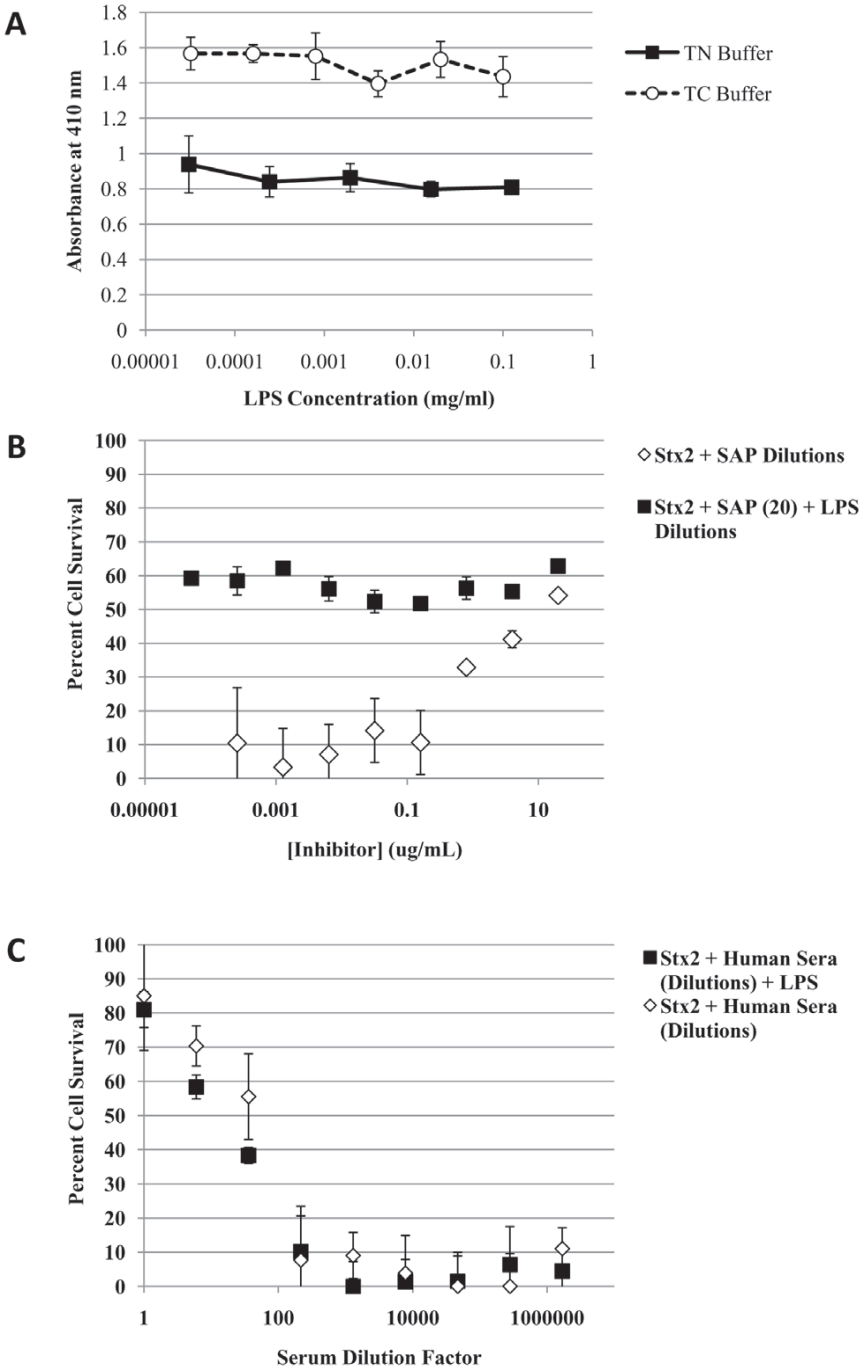
Effect of LPS on HuSAP-Stx2 interaction. **A**. The ability of HuSAP to bind to Stx2-coated EIA plates was determined in the presence of increasing concentrations of LPS. The assay was performed in the presence (TC buffer - *dashed line*) or absence (TN buffer - *solid line*) of Ca^2+^. The error bars represent the standard deviation about the means of three independent observations. **B**. Alternatively, this was assessed using the Vero-cytotoxicity assay. HuSAP at a constant concentration of 20 µg/mL was incubated with serial dilutions of LPS prior to the addition of Stx2 at a constant concentration of 2.5 ng/mL (*filled black squares*) with subsequent addition to confluent Vero cell monolayers. For comparison, Stx2 at a constant concentration of 2.5 ng/mL was also incubated with serial dilutions of HuSAP (*open diamonds*) alone before adding the mixtures to confluent Vero cell monolayers. The highest concentration of HuSAP tested in these serial dilutions was 20 µg/mL. Vero cell survival was assessed after 72 h by recording absorbance at 620 nm. The x-axis refers to the concentration of HuSAP (*open diamonds*) or LPS (*black squares*) **C**. To ensure that LPS did not bind HuSAP in complex with additional serum proteins, 100 µg/mL of LPS was pre-incubated for 30 minutes with serial dilutions of human serum (containing HuSAP) prior to adding Stx2 and subsequent addition to Vero cells. The error bars represent the standard deviation about the means of three independent observations.

HuSAP binds the majority of its ligands, perhaps including LPS [35], in a Ca^2+^-dependent manner at binding sites on the pentamer's ‘B’ face [6]. In contrast, HuSAP binds Stx2 in a unique Ca^2+^-independent manner at an unidentified binding site [8]. Since we previously demonstrated that the Ca^2+^-dependent and Stx2 HuSAP binding sites likely overlap [36], we examined whether LPS would competitively interfere with the ability of HuSAP to neutralize Stx2. Using a solid-phase ELISA-type binding assay we show that, even at the highest concentration (100 µg/mL), LPS does not prevent the interaction between Stx2 and HuSAP either in the presence or absence of Ca^2+^ (Figure 3A). We also found that LPS had no effect on the ability of HuSAP to neutralize Stx2 in a Vero cytotoxicity assay (Figure 3B), even in the presence of other serum proteins that may be required for the interaction between HuSAP and LPS (Figure 3C) [37], [38]. Therefore LPS did not appear to directly prevent HuSAP binding to Stx2.

**Figure 4:**
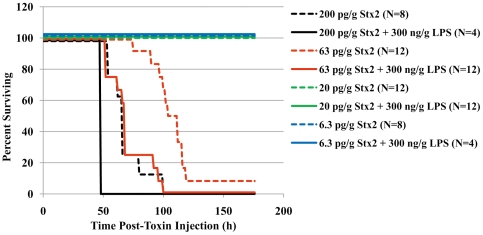
Effect of LPS on sensitivity to Stx2 in WT mice. WT mice were IP injected with 200 pg/g (*black lines*), 63 pg/g (*red lines*), 20 pg/g (*green lines*), or 6.3 pg/g BW (*blue lines*) of Stx2 in the presence (*solid lines*) or absence (*dashed lines*) of 300 ng/g BW LPS and survival was monitored for seven days. The number of animals in each group is listed beside each label.

Sugatani *et al.* previously reported that LPS reduces the minimum lethal dose of Stx2 in mice, but this observation has not been reproduced by others [23], [39]. Increased sensitivity to Stx2 independent of HuSAP+, as indicated by a lower minimum lethal dose in WT mice, could provide a possible explanation for the observed increased sensitivity to Stx2 in HuSAP+ mice. We therefore assessed the sensitivity of WT mice to a dose range of Stx2 with and without LPS treatment. As indicated by the data presented in Figure 4, no difference was found between the proportion of WT mice ultimately surviving a challenge of 63 pg/g BW Stx2 injected with or without LPS. However, at this dose of Stx2, co-administering LPS resulted in a shorter survival time (compare dashed and solid red lines Figure 4, Log Rank test p<0.001) with the average survival time being 70.3+/−16.9 h when Stx2 was injected with LPS compared to 102.7+/−13.3 h without LPS. Stx2 in combination with LPS therefore causes more rapid pathological changes than Stx2 alone.

### Expression of immune response genes in Stx2/LPS-treated mice

**Figure 5:**
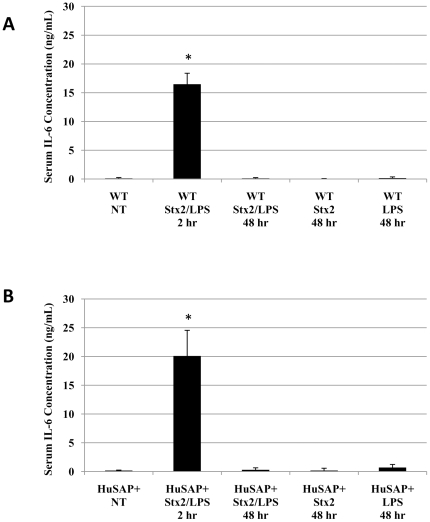
Systemic cytokine response to Stx2 and LPS. The presence of the pro-inflammatory cytokine IL-6 in the serum of mice challenged with 225 pg/g BW Stx2 and 300 ng/g BW LPS, 225 pg/g Stx2 alone or 300 ng/g LPS alone was quantified using a capture-ELISA technique (BD IL-6 OptEIA). Cytokine production in WT (**A**) and HuSAP+ (**B**) mice was compared and the immediate (2 hours) and delayed (48 hours) response was measured. The error bars represent the standard deviation about the means of three independent observations for three individual mice. Asterisks (*) denote a statistically significant (Student's *t*-test p<0.005) difference relative to WT/NT (**A**) or HuSAP+/NT (**B**).

**Figure 6:**
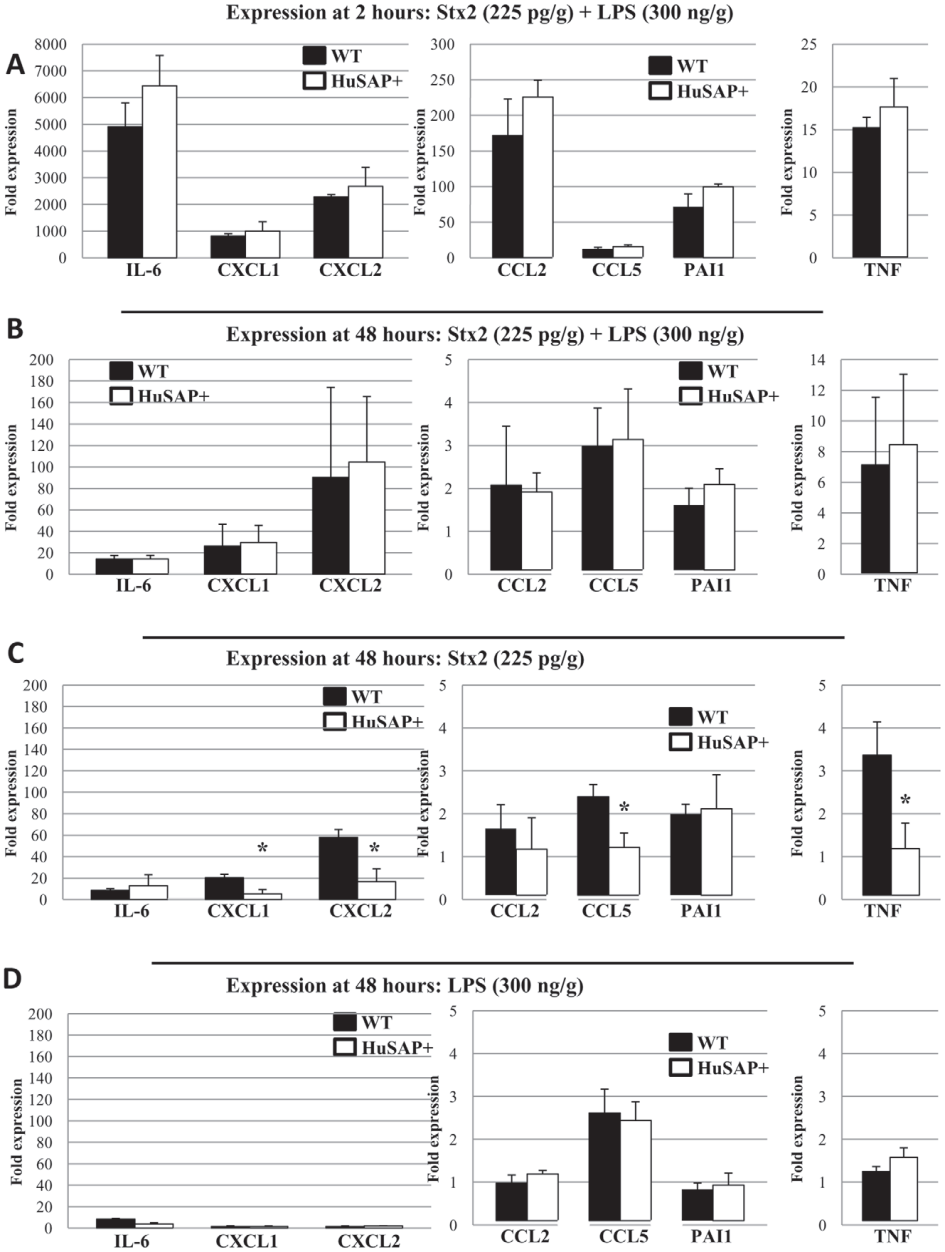
Inflammatory gene expression in kidneys of mice challenged with Stx2 and LPS. WT (*black bars*) and HuSAP+ (*white bars*) mice were challenged with 225 pg/g BW Stx2 and 300 ng/g BW LPS (**A**, **B**), 225 pg/g Stx2 alone (**C**) or 300 ng/g LPS alone (**D**). Mice were euthanized at 2 hours (**A**) or 48 hours (**B**, **C**, **D**) after toxin injection for renal gene expression analysis. Error bars represent standard deviation between mice in the same group (n = 3). Asterisks (*) in panel **C** (Stx2 alone) indicate a statistically significant difference in gene expression in HuSAP+ mice relative to similarly treated WT mice (compare white and black bars, Student's *t*-test p<0.05). No statistically significant differences between HuSAP+ and WT mice were detected in the basal expression of any gene. For clarity, expression data are not presented for mice treated with Stx2 alone after 2 hours (no statistically significant increase in expression of any genes relative to untreated animals) and LPS alone after 2 hours (no statistically significant differences relative to Stx2/LPS treatment).

To better understand the specific contribution of LPS to Stx2-mediated pathogenesis in HuSAP+ mice, we used qPCR to evaluate changes in immune response gene expression in renal tissue obtained at 2 and 48 h from HuSAP+ and WT mice challenged with Stx2 in the presence and absence of LPS. As an indicator of the systemic immune response, we also determined the serum concentrations of IL-6. The resulting data are summarized in Figures 5 and 6 and Table S2. A profound pro-inflammatory response was detected in the renal tissue immediately (2 hours) following a combined injection of Stx2 and LPS. An extremely high serum concentration of IL-6, an acute phase reactant and pro-inflammatory cytokine, was detected (Figure 5) and IL-6 gene expression in renal tissue increased a minimum of 5000 to 7000-fold. Another mediator of systemic inflammation, tissue necrosis factor α (TNFα), exhibited a more modest up-regulation (15 to 20-fold) in the kidneys of challenged mice compared to untreated animals. These changes were associated with a substantial increase in the renal expression of several other inflammatory genes. Expression of the neutrophil chemoattractant genes, CXCL1/KC and CXCL2/MIP2 (macrophage inflammatory protein 2), increased 800 to 1000-fold and 2200 to 2700-fold respectively (Figure 6A). CXCL1 and CXCL2, were recently shown to be essential for neutrophil recruitment which is key to acute renal failure developing in Stx2-challenged LPS-treated mice [24]. More moderate increases in immune response gene expression were observed for other inflammatory mediators such as CCL2/MCP-1 (monocyte-chemoattractant protein, 170 to 230-fold) and CCL5/RANTES (12 to 16-fold), both monocyte chemoattractants (Figure 6A). Expression of both these chemokines was also shown to be essential for macrophage recruitment to the kidney in Stx2-challenged LPS-treated mice and apparently play a major role in the observed renal damage [22]. Finally, although histopathological examination (Figure 2B/C) did not reveal any signs of fibrin deposition or thrombosis formation in the kidneys of these mice, we did observe a substantial increase in expression of the plasminogen activator inhibitor 1 gene (PAI-1, 70 to 100-fold), a major inhibitor of fibrinolysis pathways, which could result in a greater deposition of fibrin-clots throughout the body. Expression of several other genes (including VCAM-1, tissue factor (TF), Gb_3_ synthase and others) was also studied, and the resulting data are summarized in Table S2.

Importantly, changes in gene expression observed 2 h following toxin injection appeared to be exclusively mediated by LPS because, after 2 h, we detected no increase in gene expression above baseline in animals after injecting Stx2 alone (data not shown). Since none of the mice treated with LPS alone succumbed whereas all WT mice treated with Stx2 did, the early expression of pro-inflammatory factors does not correlate with morbidity. In addition, we observed no significant difference at 2 h between immune response gene expression in WT relative to HuSAP+ mice, presumably because the response was mediated exclusively by LPS and providing evidence that HuSAP does not antagonize LPS activity *in vivo*, despite previous reports indicating that it does [35].

After 48 hours, no IL-6 was detected in the serum of Stx2- and LPS-challenged mice (Figure 5) and only a mild up-regulation in kidney IL-6 expression was observed (approximately 0.25% of the expression measured at 2 hours). For the majority of inflammatory genes, the magnitude of expression at 48 h was substantially reduced relative to the 2 h values (note the change in y-axis scale in Figure 6B compared to 6A). The only exception was TNFα expression, which remained relatively constant at 2 and 48 hours. Notably, the increased TNFα gene expression at 48 hours appeared to be mediated almost exclusively by Stx2 (Figure 6C/D). Moderate up-regulation of the IL-6 (8.4-fold+/−1.6), CXCL1 (20.3-fold+/−3.2), and CXCL2 (57.9-fold+/−7.3) genes was observed in WT mice injected with Stx2 alone. Nearly identical changes (IL-6 [14.3-fold+/−3.3], CXCL1 [26.3-fold+/−20.3], and CXCL2 [90.2-fold+/−84.1]) were observed in WT mice injected with both Stx2 and LPS (Figure 6B). Stx2 alone also caused small, but significant, up-regulation of CCL5 and PAI-1 gene expression (Figure 6C). At 48 h, we detected almost no changes in gene expression in mice injected with LPS alone relative to untreated mice (Figure 6D). Importantly, the 48 h profile of immune response gene expression induced by Stx2 alone allowed us to detect differences which were consistent with the survival of WT and HuSAP+ mice. Significant differences in TNFα (64.66% less expression, p<0.05, Student's *t*-test), CXCL1 (74.15% less expression, p<0.05, Student's *t*-test), CXCL2 (71.3% less expression, p<0.05, Student's *t*-test), and CCL5 (49.4% less expression, p<0.05, Student's *t*-test) gene expression were observed between HuSAP+ mice relative to their WT litter-mates in response to Stx2 alone (Figure 6C). This reduced expression of these inflammatory genes is associated with the survival of HuSAP+ mice following challenge by Stx2 alone (Figure 1) and therefore strongly suggests that increased expression of these four genes is essential for mediating the acute renal failure and mortality observed in WT mice following a challenge of Stx2 alone.

Of these genes, administering LPS alone caused significant up-regulation of only CCL5 (2.61-fold+/−0.56, Student's *t*-test p = 0.048) at 48 h and no significant up-regulation of CXCL1 (1.52-fold+/−0.49) or CXCL2 (1.47-fold+/−0.44) gene expression. Despite the observation that LPS by itself had no apparent effect on gene expression, it appeared to enhance inflammatory gene expression induced by Stx2 in HuSAP+ mice. For each of these genes, injecting Stx2 combined with LPS resulted in an identical up-regulation of inflammatory gene expression in HuSAP+ relative to WT mice (Figure 6B). This observation further underscores the relationship between CXCL1, CXCL2 and CCL5 gene expression and survival since both HuSAP+ and WT mice injected with Stx2 and LPS exhibited enhanced expression of these three genes after 48 h and ultimately succumbed (Figure 1).

### Inhibition of Immune Response

**Figure 7:**
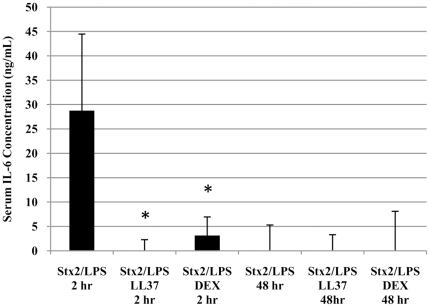
Systemic cytokine response in HuSAP+ mice to Stx2 and LPS in the presence of inhibitor. HuSAP+ mice were challenged with 225 pg/g BW Stx2 and 300 ng/g BW LPS alone, admixed with 0.5 µg/g BW antimicrobial peptide LL-37 or 30 minutes after IP injection of 2.5 µg/g BW dexamethasone (DEX). The immediate (2 hours) and delayed (48 hours) concentration of IL-6 was monitored. The error bars represent the standard deviation about the means of three independent observations for three individual mice. Asterisks (*) denote a statistically significant difference relative to mice treated with Stx2/LPS in the absence of inhibitor at the same time post-toxin injection (Student's *t*-test p<0.005).

**Figure 8:**
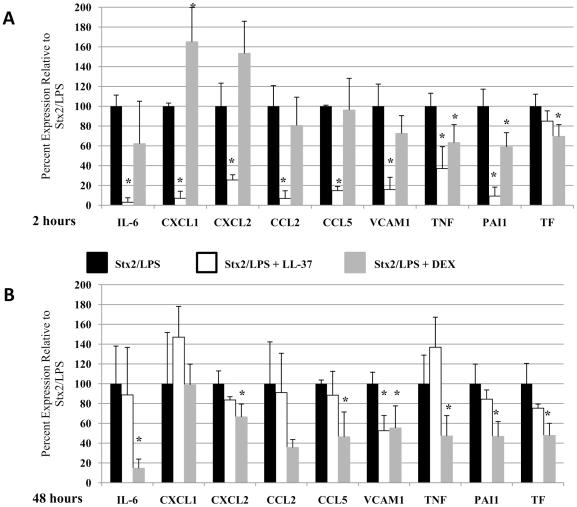
Inflammatory gene expression in kidneys of HuSAP+ mice challenged with Stx2 and LPS in the presence of inhibitor. HuSAP+ mice were challenged with 225 pg/g BW Stx2 and 300 ng/g BW LPS alone (*black bars*), admixed with 0.5 µg/g BW antimicrobial peptide LL-37 (*white bars*) or 30 minutes after subcutaneous injection of 2.5 µg/g BW dexamethasone (*grey bars*). Mice were euthanized at 2 hours (**A**) or 48 hours (**B**) after toxin injection for renal gene expression analysis. Fold expression was normalized to the no treatment group (not shown) and the data are presented as relative (%) to the gene expression observed in the Stx2/LPS group. Error bars represent standard deviation between mice in the same group (n = 3). Asterisks (*) indicate a statistically significant difference in gene expression compared to mice treated with Stx2/LPS without inhibitor (*black bars*) at the same time post-toxin injection (Student's *t*-test p<0.05).

We next tested two anti-inflammatory compounds for their ability to provide therapeutic benefit in LPS-sensitized Stx2-treated HuSAP+ mice. The two agents were selected for their indirect (dexamethasone) and combined direct/indirect (LL-37) inhibition of the effects of LPS. As a result of these differences in their mode of action, the two agents had distinct effects on the inflammatory response initiated by Stx2 and LPS in HuSAP+ mice (Figures 7 and 8). Except for TF, LL-37 inhibited the 2 h response of all the other inflammatory genes by 63 to 97% (Figure 8A). The LL-37 treatment also resulted in a reduction in the serum concentration of IL-6 to a sub-detectable amount (Figure 7). In contrast, DEX had little impact on the 2 h immune response, significantly reducing only the expression of TNFα, PAI1, and TF (Figure 8A) and the serum concentration of IL-6 (Figure 7). For CXCL1, DEX treatment caused a significant increase in gene expression after 2 h. This trend, however, was reversed at 48 h. Despite the profound reduction in the immediate inflammatory response, LL-37 failed to reduce the expression of any of the genes after 48 h, with the exception of VCAM1 (Figure 8B). Conversely, and despite its minimal effect on the immediate response, DEX treatment significantly reduced the expression of CXCL2, CCL5, TNFα, VCAM1, PAI1 and TF by 35 to 85% after 48 hours (Figure 8B). CXCL2, CCL5, and TNFα were also reduced in HuSAP+ mice (relative to WT) 48 hours after Stx2 treatment (Figure 6C) and therefore may be crucial for the inflammatory pathology leading to acute renal failure.

**Figure 9:**
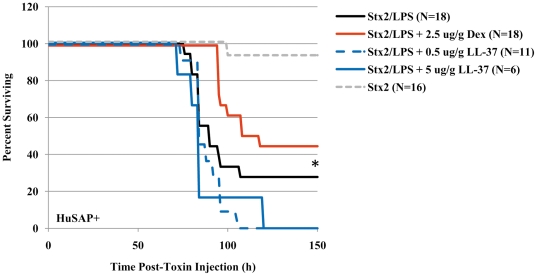
Survival of HuSAP+ mice challenged with Stx2 and LPS in the presence of inhibitor. HuSAP+ mice were challenged with 225 pg/g BW Stx2 and 300 ng/g BW LPS alone (*black line*), admixed with 0.5 µg/g BW antimicrobial peptide LL-37 (*blue dashed line*), admixed with 5.0 µg/g BW LL-37 *(solid blue line)* or 30 minutes after subcutaneous injection of 2.5 µg/g BW dexamethasone (*solid red line*). HuSAP+ mice challenged with 225 pg/g BW Stx2 alone were included for comparison (*grey dashed line*). The number of animals in each group is listed beside each label. The asterisks (*) denotes that the differences in survival between HuSAP+ mice treated with Stx2/LPS without inhibitor and those treated with Stx2/LPS and dexamethasone was statistically significant (Log Rank test p = 0.048).

The well-defined differences in the effects of LL-37 and DEX on the immune response allowed us to relate specific changes in gene expression to survival in LPS-sensitized Stx2-treated mice. As indicated by the data presented in Figure 9, only DEX protected HuSAP+ mice challenged with the combination of Stx2 and LPS (compare red and black lines Figure 9, Log Rank test p = 0.048). In contrast to DEX, LL-37 treatment failed to provide any protective benefit (compare dashed blue and black lines Figure 9), even at an extremely high dose (5 µg/g BW, solid blue line, Figure 9). Despite the fact that LL-37 drastically reduced the serum cytokine concentration (Figure 7) and inhibited the strong up-regulation of inflammatory genes that was induced by LPS in the kidneys 2 h after toxin injection (Figure 8), it had no effect on mouse survival. This again emphasized the importance of sustained as opposed to acute inflammation in the kidneys, regardless of the magnitude of differences in gene expression. Similar to the HuSAP-mediated protection, decreased sensitivity to toxin in the presence of DEX was associated with the renal production of chemokines of the CXC and CC families, as well as the systemic inflammatory mediator TNFα, 48 h after toxin injection.

## Discussion

The loss of intestinal barrier function during EHEC infection is thought to contribute to direct entry of Stx into the circulation. This would presumably also allow entry of bacterial endotoxin into the circulation, although there is limited evidence this occurs following EHEC infection. LPS has been detected in the serum of HUS patients following *S. dystenteriae* 1 infection, but not EHEC infection [40], [41]. In one study, O157 LPS was detected on the surface of platelets in EHEC-infected HUS patients, but not in patients that did not develop HUS [42]. Circulating antibodies specific to O157 LPS are also common in HUS patients and serum LPS binding protein (LBP) concentrations are increased [43], [44], [45]. The simple presence of antibodies to LPS in circulation does not predict progression to HUS in EHEC-infected patients [46], but these studies all suggest that at least some LPS has entered the circulation in these subjects. In some of the more severe cases of HUS preceded by EHEC infection, subjects were observed to exhibit many of the clinical signs associated with septic shock, suggesting severe endotoxemia [47]. There is abundant evidence that an inflammatory response occurs in patients following EHEC infection which could be the result of a mild endotoxemia. The evidence presented herein suggests that HuSAP is unable to prevent renal damage in mice in the presence of a prolonged inflammatory response.

The role LPS plays in HUS has not been clearly established, in part because of apparent differences in its role in various animal models. In the present study we demonstrate that LPS accelerates the fatal pathological process initiated by Stx2 in mice (Figure 4). Several studies have reported similar results although in certain animal models, LPS is able to both enhance and prevent Stx toxicity [48]. For example, Ikeda *et al.* previously reported that co-administering LPS with Stx2 caused haematological and histological alterations in mice consistent with HUS but when LPS was injected 24 hours before Stx2, neither of these effects were observed [49]. LPS is apparently protective when injected 18–96 hours before Stx, but not when injected within one hour of Stx or after Stx [34], [39], [49]. These differences correlate with the occurrence of pro-inflammatory cytokines (early response) and anti-inflammatory cytokines (late response) in circulation after LPS injection and, as a result, it has been proposed that the intensity and duration of these responses might explain differences in the severity of the symptoms [34]. We now show, however, that reducing the pro-inflammatory response (Figure 7 and 8A) which occurs immediately after Stx2/LPS injection using LL-37 had no impact on the ultimate survival outcome (Figure 9). In contrast, inhibition of the sustained pro-inflammatory response by HuSAP or DEX (Figures 5 and 7) was protective.

DEX protection was specifically associated with a reduced expression of CXC and CC-family chemokines, which are responsible for recruiting neutrophils and macrophages to inflamed tissues. Antibody neutralization of these chemokines was previously shown to limit recruitment of inflammatory cells to the kidneys following Stx2 and LPS challenge and thereby lessen renal damage, but there was no survival benefit to separately blocking each family [22], [24]. Reducing both CC and CXC chemokine families simultaneously, which was achieved herein, may therefore be necessary for protection. The additional impact of reducing TNFα expression may also play a significant role in protection since TNFα has been demonstrated to up-regulate endothelial cell Gb_3_ expression, increase Stx *in vitro* cytotoxicity, and participate in TF up-regulation by Stx, in addition to its numerous pro-inflammatory effects [50], [51], [52]. Recently, TNFα was implicated by Lentz *et al.* as a key mediator of renal tubule epithelial cell apoptosis, renal failure and lethality following Stx1 intoxication [53]. Notably, TNFα expression is localized to only the kidneys in mice challenged with Stx and high concentrations are detected in the urine, as opposed to the serum, of children in the acute phase of EHEC-associated HUS, both of which suggest that TNFα's primary role is in mediating the kidney-specific inflammatory response [17], [54].

Although we have not extensively explored mechanisms of HUS coagulopathy in the current report, it is possible that the primary action of LPS is to induce a pro-thrombotic state. Stahl and colleagues recently reported that O157 LPS binds and activates human and murine platelets [42] and that Stx2 and *E. coli* LPS synergize to produce monocyte-platelet and neutrophil-platelet aggregates and stimulate tissue factor (TF) release into plasma [55]. Our data also suggest a role for the coagulation cascade in pathogenesis, since Stx2/LPS induce renal expression of plasminogen-activator inhibitor 1 (PAI-1) and TF (Figure 6 and Table S2) and repression of these genes is associated with survival. TF up-regulation at 48 hours is significantly reduced in HuSAP+ relative to WT mice (Student's *t*-test p = 0.02, Table S2), and DEX inhibits the expression of both these pro-thrombotic factors at 2 and 48 hours after toxin injection (Figure 8).

Since HuSAP effectively neutralizes Stx2 in the absence of LPS, we propose that neutralizing the effects of LPS may be an effective therapeutic approach in EHEC-infected patients who already possess HuSAP in their serum [8]. Herein we investigated two possible LPS-inhibitors that act through two diverse mechanisms. Human cathelicidin-derived cationic antimicrobial peptide LL-37 modulates the LPS-induced inflammatory response and is capable of preventing endotoxemia/sepsis in a variety of animal models, including the mouse [56], [57]. It does so *via* a complex mechanism involving both direct and indirect intervention. LL-37 blocks LPS from binding LPS-binding protein (LBP) and prevents its subsequent interaction with both the soluble and membrane-bound form of CD14 on the surface of several cell types thereby preventing Toll-like receptor activation [58]. As well, LL-37 alters intracellular signal transduction pathways, selectively modulates pro- and anti-inflammatory gene transcription and is thought to influence protein translation or stabilization [31].

DEX, in contrast, is an anti-inflammatory drug of the synthetic glucocorticoid class and is used extensively in clinical practice in managing inflammatory and autoimmune conditions [59], [60]. Although the exact mechanism of action is unknown, DEX acts on a variety of immune cell types, including macrophages, neutrophils and epithelial cells, and suppresses the production of pro-inflammatory cytokines [32], [33]. Specifically, DEX protects mice against high dose-LPS mediated lethality by reducing TNFα and IL-6 expression through a variety of intracellular signalling pathways [33]. In addition, DEX suppression of the inflammatory response enhances the survival of mice challenged with Stx2 alone [34], [61], [62]. These studies and ours suggest the potential for DEX as a therapeutic agent in HUS. In this regard, DEX is an effective treatment for two similar conditions, idiopathic thrombocytopenic purpura (ITP) and non-EHEC-associated thrombotic thrombocytopenic purpura (TTP), and is therefore worth consideration as part of the management of EHEC-infected HUS patients [59], [63].

## Supporting Information

Table S1 **The primers used for amplification and references for each selected gene are summarized.**
(DOCX)

Table S2 **Additional gene expression in kidneys of mice challenged with Stx2 and LPS.** As in Figure 6, WT and HuSAP+ mice were challenged with 225 pg/g BW Stx2 and 300 ng/g BW LPS, 225 pg/g Stx2 alone or 300 ng/g LPS alone. Mice were euthanized at 2 hours or 48 hours after toxin injection for renal gene expression analysis. Asterisks (*) indicate a statistically significant difference in gene expression in HuSAP+ mice relative to similarly treated WT mice (Student's *t*-test p<0.05). No statistically significant differences between HuSAP+ and WT mice were detected in the basal expression of any gene. ND indicates that these genes were not tested in these groups.
(DOCX)
