# Supplementary material for: Lipopolysaccharide Renders Transgenic Mice Expressing Human Serum Amyloid P Component Sensitive to Shiga Toxin 2
Source: PLoS One. 2011 Jun 24;6(6):e21457. doi: 10.1371/journal.pone.0021457 (PMC3123346; doi:10.1371/journal.pone.0021457)
Supplement: Table S1 — The primers used for amplification and references for each selected gene are summarized. (DOCX) [file pone.0021457.s001.docx]

**Table S1**

| Gene | Symbol | Primers (5`-3’) | Anneal (°C) | Efficiency | Reference |
| --- | --- | --- | --- | --- | --- |
| Beta-actin | *ActB* | F: AGATCATGTTTGAGACCTTCA  R: TCGTAGATGGGCACAGTGT | 58 | 1.890 | This study |
| Chemokine C-X-C ligand 1 | *Cxcl1* | F: AGCCACCCGCTCGCTTCTCT  R: CAAGGCAAGCCTCGCGACCA | 60 | 1.909 | 1 |
| Chemokine C-X-C ligand 2 | *Cxcl2* | F: ACCACCAGGCTACAGGGGCT  R: TCCTGGGGGCGTCACACTCA | 60 | 1.784 | 1 |
| Chemokine C-C ligand 5 (RANTES) | *Ccl5* | F: TCGTGCCCACGTCAAGGAGTATTT  R: ACTAGAGCAAGCGATGACAGGGAA | 62 | 1.990 | 2 |
| Gb3 synthase | *Gb3s* | F: ATCGCACTCCTCTGGAAGTTTGGT  R: ACAGTGCCAAGAACTCATGCTTGC | 60 | 1.870 | This study |
| Glyceraldehyde-3-P dehydrogenase | *Gapdh* | F: TGTGTCCGTCGTGGATCTGA  R: TTGCTGTTGAAGTCGCAGGAG | 60 | 1.888 | 3 |
| Interleukin 6 | *Il6* | F: ATCCAGTTGCCTTCTTGGGACTGA  R: TAAGCCTCCGACTTGTGAAGTGGT | 60 | 1.98 | 4 |
| Macrophage inflammatory protein-1 alpha (CCL3) | *Mip-1α* | F: TGTTTGCTGCCAAGTAGCCACATC  R: AACAGTGTGAACAACTGGGAGGGA | 60 | 1.845 | 2 |
| Monocyte chemoattractant protein-1 (CCL2) | *Mcp-1* | F: TTGACCCGTAAATCTGAAGC  R: CGAGTCACACTAGTTCACTG | 60 | 1.981 | 2 |
| Plasminogen activator inhibitor-1 | *Pai-1* | F: GACACCCTCAGCATGTTCATC  R: AGGGTTGCACTAAACATGTCAG | 64 | 1.879 | 5 |
| Tissue factor | *Tf* | F: ATGTGACCTGGGCCTATGAA  R: TTACTGGCTGTCCGAGGTTT | 60 | 1.869 | 6 |
| Transforming growth factor beta 1 | *Tgfβ1* | F: TGACGTCACTGGAGTTGTACGG  R: GGTTCATGTCATGGATGGTGC | 60 | 1.806 | 7 |
| Tumour necrosis factor | *Tnf-α* | F: CATCTTCTCAAAATTCGAGTGACAA  R: TGGGAGTAGACAAGGTACAACCC | 60 | 1.851 | 7 |
| Vascular cell adhesion molecule-1 | *Vcam-1* | F: TGACAAGTCCCCATCGTTGA  R: ACCTCGCGACGGCATAATT | 60 | 2.010 | 8 |

1. Rozen, S. & Skaletsky, H.J. (2000). Primer3 on the WWW for general users and for general programmers. *Methods in Molecular Biology, 132*, 365-86.
2. Keepers, T.R., Gross, L.K., & Obrig, T.G. (2007). Monocyte chemoattractant protein 1, macrophage inflammatory protein 1α, and RANTES recruit macrophages to the kidney in a mouse model of hemolytic-uremic syndrome. *Infection and Immunity, 75*, 1229-1236.
3. Kondo, R., Higuchi, M., Takahashi, M., Oie, M., Tanaka, Y., Gejyo, F., & Fujii, M. (2006). Human T-cell leukemia virus type 2 Tax protein induces interleukin 2-independent growth in a T-cell line. *Retrovirology, 3*, 88.
4. Hollingshead, H.E., Morimura, K., Adachi, M., Kennett, M.J., Billin, A.N., Willson, T.M., Gonzalez, F.J., & Peters J.M. (2007). PPARbeta/delta protects against experimental colitis through a ligand-independent mechanism. *Digestive Diseases and Sciences, 52*, 2912-9.
5. Mutoh, M., Niho, N., Komiya, M., Takahashi, M., Ohtsubo, R., Nakatogawa, K., Ueda, K., Sugimura, T., & Wakabayashi, K. (2008). Plasminogen activator inhibitor-1 (Pai-1) blockers suppress intestinal polyp formation in Min mice. *Carcinogenesis, 29*, 824-9.
6. Furnkranz, A., Schober, A., Bochkov, V.N., Bashtrykov, P., Kronke, G., Kadl, A., Binder, B.R., Weber, C., & Leitinger, N. (2005). Oxidized phospholipids trigger atherogenic inflammation in murine arteries. *Arteriosclerosis, Thrombosis, and Vascular Biology, 25*, 633-8.
7. Guilietti, A., Overbergh, L., Valckx, D., Decallonne, B., Bouillon, R., & Mathieu, C. (2001). An overview of real-time quantitative PCR: applications to quantify cytokine gene expression. *Methods, 25*, 386-401.
8. Hosking, B.M., Wang, S.C., Downes, M., Koopman, P., & Muscat, G.E. (2004). The VCAM-1 gene that encodes the vascular cell adhesion molecule is a target of the Sry-related high mobility group box gene, Sox18. *The* *Journal of Biological Chemistry, 279*, 5314-22.
