# Supplementary material for: Lipopolysaccharide Renders Transgenic Mice Expressing Human Serum Amyloid P Component Sensitive to Shiga Toxin 2
Source: PLoS One. 2011 Jun 24;6(6):e21457. doi: 10.1371/journal.pone.0021457 (PMC3123346; doi:10.1371/journal.pone.0021457)
Supplement: Table S2 — Additional gene expression in kidneys of mice challenged with Stx2 and LPS. As in Figure 6, WT and HuSAP+ mice were challenged with 225 pg/g BW Stx2 and 300 ng/g BW LPS, 225 pg/g Stx2 alone or 300 ng/g LPS alone. Mice were euthanized at 2 hours or 48 hours after toxin injection for renal gene expression analysis. Asterisks (*) indicate a statistically significant difference in gene expression in HuSAP+ mice relative to similarly treated WT mice (Student's t-test p<0.05). No statistically significant differences between HuSAP+ and WT mice were detected in the basal expression of any gene. ND indicates that these genes were not tested in these groups. (DOCX) [file pone.0021457.s002.docx]

**Table S2**

| Treatment | Mouse Strain | Expression relative to “no treatment” | | | | |
| --- | --- | --- | --- | --- | --- | --- |
|  |  | ***Tf*** | ***Gb3s*** | ***Vcam-1*** | ***Tgfβ1*** | ***Mip-1α*** |
| No treatment | WT | 1.00±0.14 | 1.01±0.25 | 1.00±0.23 | 1.03±0.38 | 1.45±1.57 |
|  | HuSAP+ | 0.87±0.13 | 1.21±0.02 | 0.69±0.13 | 1.01±0.17 | 1.40±1.43 |
| Stx2/LPS 2 h | WT | 1.46±0.16 | 0.38±0.08 | 15.47±1.22 | 1.18±0.22 | 43.21±11.29 |
|  | HuSAP+ | 1.84±0.23 | 0.41±0.03 | 15.24±3.33 | 1.84±0.25 | 47.60±5.50 |
| Stx2/LPS 48 h | WT | 1.69±0.16 | 0.61±0.09 | 0.81±4.42 | ND | ND |
|  | HuSAP+ | 1.83±0.25 | 0.63±0.03 | 0.80±4.60 | 1.57±0.13 | 1.64±0.46 |
| Stx2 48 h | WT | 2.14±0.31 | 0.67±0.03 | 0.87±0.78 | ND | ND |
|  | HuSAP+ | 1.49±0.35* | 0.77±0.22 | 0.50±0.59 | ND | ND |
| LPS 48 h | WT | 0.83±0.07 | 0.60±0.14 | 0.85±0.12 | ND | ND |
|  | HuSAP+ | 0.75±0.7 | 0.74±0.07 | 0.65±0.22 | ND | ND |
